# An algorithm to identify cases of pulmonary arterial hypertension from the electronic medical record

**DOI:** 10.1186/s12931-022-02055-0

**Published:** 2022-05-28

**Authors:** Kyle P. Schuler, Anna R. Hemnes, Jeffrey Annis, Eric Farber-Eger, Brandon D. Lowery, Stephen J. Halliday, Evan L. Brittain

**Affiliations:** 1 Department of Internal Medicine, Vanderbilt University Medical Center, Nashville, TN USA; 2 Division of Allergy, Pulmonary and Critical Care Medicine, Vanderbilt University Medical Center, Nashville, TN USA; 3 Division of Cardiovascular Medicine, Vanderbilt Pulmonary Circulation Center, 2525 West End Avenue, Nashville, TN USA; 4 Vanderbilt Institute for Clinical and Translational Research (VICTR), Nashville, TN USA; 5 Division of Pulmonary and Critical Care Medicine, University of Wisconsin School of Medicine and Public Health, Madison, WI USA

**Keywords:** Pulmonary hypertension, Machine learning, Algorithm

## Abstract

**Background:**

Study of pulmonary arterial hypertension (PAH) in claims-based (CB) cohorts may facilitate understanding of disease epidemiology, however previous CB algorithms to identify PAH have had limited test characteristics. We hypothesized that machine learning algorithms (MLA) could accurately identify PAH in an CB cohort.

**Methods:**

ICD-9/10 codes, CPT codes or PAH medications were used to screen an electronic medical record (EMR) for possible PAH. A subset (Development Cohort) was manually reviewed and adjudicated as PAH or “not PAH” and used to train and test MLAs. A second subset (Refinement Cohort) was manually reviewed and combined with the Development Cohort to make The Final Cohort, again divided into training and testing sets, with MLA characteristics defined on test set. The MLA was validated using an independent EMR cohort.

**Results:**

194 PAH and 786 “not PAH” in the Development Cohort trained and tested the initial MLA. In the Final Cohort test set, the final MLA sensitivity was 0.88, specificity was 0.93, positive predictive value was 0.89, and negative predictive value was 0.92. Persistence and strength of PAH medication use and CPT code for right heart catheterization were principal MLA features. Applying the MLA to the EMR cohort using a split cohort internal validation approach, we found 265 additional non-confirmed cases of suspected PAH that exhibited typical PAH demographics, comorbidities, hemodynamics.

**Conclusions:**

We developed and validated a MLA using only CB features that identified PAH in the EMR with strong test characteristics. When deployed across an entire EMR, the MLA identified cases with known features of PAH.

**Supplementary Information:**

The online version contains supplementary material available at 10.1186/s12931-022-02055-0.

## Introduction

Pulmonary arterial hypertension (PAH) is a progressive and fatal rare disease with an incidence of approximately 1/1,000,000 population. PAH is a challenging diagnosis to make often resulting in delayed diagnosis and etiology is unknown in many cases [1]. Much of our current understanding of disease epidemiology has come from carefully performed registries including patients evaluated at expert centers [2–4]. However, these registries have inadequate ability to identify risk factors that precede disease development, clinical features that may be clues to disease presence and facilitate earlier diagnosis, or real-world efficacy data on PAH therapies. The ability to confidently identify PAH cases in large administrative claims datasets and electronic medical records (EMRs) is a key first step to answering these impactful questions. Limiting algorithm components only to administrative data is advantageous because extraction of raw diagnostic data (e.g. hemodynamics) is not practical in most EMR and such data are not available in claims databases. Application of machine learning approaches that automatically weight different components to increase accuracy may outperform the rules-based approaches published to date.

PAH is a distinct form of pulmonary hypertension (PH) which must be distinguished clinically from other forms of PH by extensive evaluation of lung and cardiac structure and function and right heart catheterization [5, 6]. PAH is thought to have a distinct molecular etiology from other forms of PH and is treated with specific medications that are not uniformly known to be beneficial in other forms, particularly in PH associated with left heart failure. These complex diagnostic and treatment requirements are coupled with imprecision in billing codes used for PAH [7], which taken together make confident identification of PAH patients within a large administrative claims database highly challenging. Prior attempts at creating algorithms have had limited diagnostic accuracy for PAH specifically to date, and reliance on administrative coding alone has proven insufficient to reliably identify PAH patients from the medical record [4, 7–14]. While machine learning algorithm has been used to identify PH in claims data [15], this approach has not, to our knowledge, specifically been used for PAH. These literature limitations are, in part, because to manual review of patient level data to adjudicate a diagnosis of PAH was limited.

We hypothesized that a three-component algorithm based on ICD codes, procedural codes for right heart catheterization, and PAH-specific medications might perform best and offer widespread applicability to most EMRs and commercially available administrative claims databases. Using a combination of well-phenotyped patients within a large de-identified electronic medical record, manual review of potential PAH cases, novel machine learning analytic techniques, and a split cohort internal in silico validation cohort, we sought to develop and test an algorithm that accurately identifies PAH patients with only administrative claims data at a large tertiary care center.

## Methods

### Study population

The Vanderbilt University Institution Review Board approved this study with a waiver of consent as non-human subjects research. Data for this study were extracted from Vanderbilt’s Synthetic Derivative (SD) database, which is a deidentified form of the EMR at Vanderbilt University Medical Center previously described [16, 17]. The SD contained > 2.5 million unique patients at the time data were extracted.

### Development cohort

**Fig. 1:**
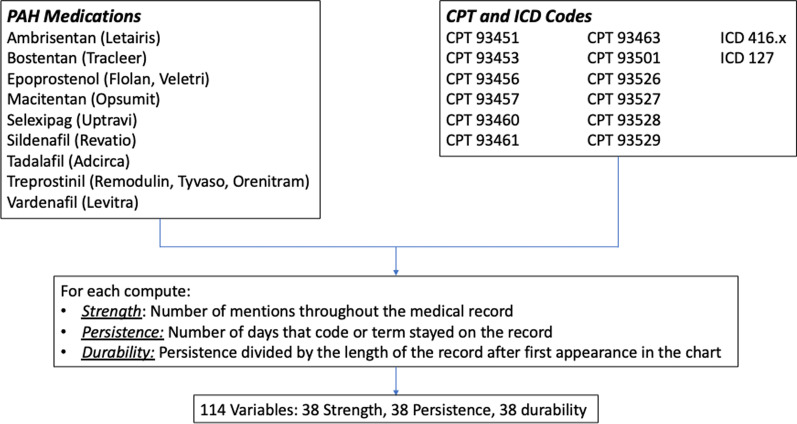
Features of screening tool. The screening tool included PAH medications, including brand and generic names, current procedural terminology (CPT) codes for right heart catheterization and International Classification of Diseases (ICD) 9 or 10 codes for pulmonary hypertension. With final testing of the algorithm, characteristics of the algorithm that were tested included the strength, persistence and durability

Our final algorithm was developed in an iterative process involving multiple stages as is standard [18]. We first identified sufficient true cases of PAH within the SD to develop an initial algorithm, which we refer to as the Development Cohort. To narrow our search, we used a screening tool in which we defined possible PAH as having at least one of the following features suggestive of a diagnosis of PAH (Fig. 1): ICD-9/10 code for primary pulmonary hypertension, CPT code variant for right heart catheterization, or PAH-specific medications (capturing both generic and brand names). The prevalence of each variable in the Synthetic Derivative is provided in Additional file 1: Table S1. This enrichment step identified 8000 charts with at least one feature, which we then sorted by the number of features above to focus the chart review on patients most likely to have PAH. Then, 980 charts with at least one feature were independently reviewed by PH specialists (KS, SH, or ARH). In addition to confirmatory chest imaging and pulmonary function testing, the determination of PAH required a right heart catheterization demonstrating a mean pulmonary artery pressure ≥ 25 mmHg, pulmonary vascular resistance ≥ 3 Wood units, and pulmonary capillary wedge pressure ≤ 15 mmHg according to the World Symposium on Pulmonary Hypertension (WSPH) guidelines at the time of patient evaluation [19]. These gold-standard PAH cases and “not PAH” controls were used for the first stage in machine learning algorithm development. The Development Cohort was randomly divided into 70% training set and 30% test set to produce our first algorithm. The algorithm resulting from the Test Set of the Development Cohort is referred to as the “Initial Algorithm” with test characteristics reported below.

### Refinement cohort

The Initial Algorithm developed from the test set of the Development Cohort was deployed against the remaining 7020 charts flagged in our screening tool to create the Refinement Cohort which contained 741 additional predicted PAH cases. Among the 741 predicted cases, we identified an additional 468 manually confirmed cases of PAH.

### Final cohort

In our final step, the Development and Refinement Cohorts were combined into a single cohort, Final Cohort, including PAH cases and non-cases from 1694 manually reviewed charts and excluding cases < 18 from the Development Cohort. The purpose of this step was to apply an additional layer of algorithm training. “This total cohort was also divided randomly and separated into a training set consisting of 80% of the cases and a test set consisting of the remaining 20% of cases.”. The results reported for our final algorithm are those developed from the test set of the Final Cohort. Although the cases in this final set are derived from the Development and Refinement Cohorts, the final model was trained anew on the Final Cohort and is independent of prior models (i.e. all model parameters were randomly set in the final model before beginning training on the Final Cohort). The algorithm evolved from the Development and Refinement Cohorts and as such this specific version of the algorithm had not previously been examined against the cases and controls.

### Internal validation

Algorithm validation is a minimum requirement of machine learning studies. Our final algorithm was deployed in the remainder of patients in the SD that were not previously studied in any Cohorts (approximately 2 million cases). The purpose of this internal validation set was to report the characteristics of patients selected by the algorithm and determine their similarity to published PAH demographics and clinical features [20–22]. This step simulates application of the algorithm in an administrative dataset because it only uses claims-based data. These cases were aggregated to identify the prevalence of commonly published features of PAH: age, sex, comorbidities including congenital heart disease and connective tissue disease by ICD9/10 codes as previously published [23], PAH-medication use, and hemodynamic data from right heart catheterization (mean right atrial pressure, mean pulmonary artery pressure, pulmonary capillary wedge pressure, cardiac output, and cardiac index). The features of cases identified in the SD were then compared with published PAH registries.

### Machine learning algorithms

We used several distinct machine learning techniques described in the supplement to identify an algorithm with the best performance characteristics: Elastic net [24], random forests (RF) [25], extreme gradient boosting (XGBoost) [26]. For optimization, we used Bayesian optimization [27]. Algorithms were trained using the ranger [28], xgboost [29], and glmnet [30] R packages. We performed cross validation using the caret [31] R package and Bayesian hyperparameter optimization using the mlrMBO [20] R package.

### Statistical analysis

Sensitivity, specificity, positive predictive value (PPV), negative predictive value (NPV), and area under the curve (AUC) of the initial algorithm were calculated from the test set of the Development Cohort and the performance of the final algorithm were calculated from the test set of the Final Cohort. The best performing algorithm (RF) was evaluated to determine which features were most important for categorization. Each feature (ICD-9/10 code for primary pulmonary hypertension, CPT code variant for right heart catheterization, or PAH-specific medications) was assigned a strength, persistence, and durability. Strength was defined as the number of mentions throughout the medical record. Persistence was defined as the number of days that code or term stayed on the record. Durability was defined as the persistence divided by the length of the record after the first appearance on the record. Statistics were computed using the same R package used to train the models, Classification And REgression Training (caret). TRIPOD checklist is in the supplemental material.

## Results

### Initial development of algorithm

**Fig. 2:**
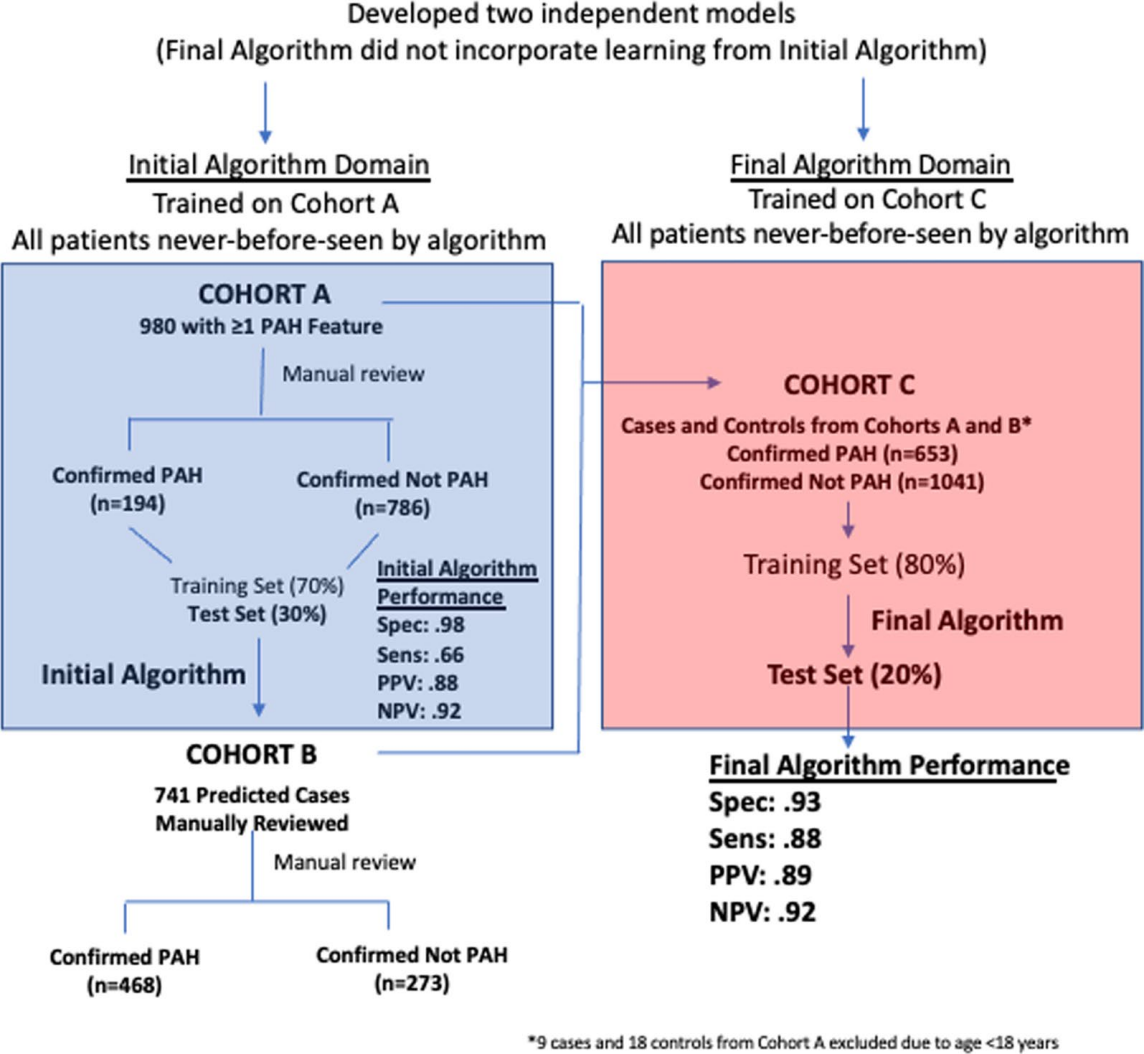
Study flow diagram. *PAH*  pulmonary arterial hypertension, *Spec*  specificity, *Sens*  sensitivity, *PPV*  positive predictive value, *NPV*  negative predictive value

Among patients in the SD flagged by the screening tool with an PAH feature, we manually reviewed 980 charts, which comprised the Development Cohort (Fig. 2). Of these, 194 were determined to have true PAH and 786 were confirmed as not PAH. Based on the Development Cohort, our machine learning algorithms (RF, XGBoost and Elastic Net) underwent initial training and testing. These 980 manually reviewed cases were divided randomly into a training set (70%) and a test set (30%). The test characteristics of the best-performing Initial Algorithm (RF) in the Development Cohort were: specificity: 0.98, sensitivity: 0.66, positive predictive value: 0.88, negative predictive value: 0.92. Results of the initial algorithm using each of the three machine learning approaches and comparison with mediation use alone are reported in Additional file 1: Table S2, Fig. S1. The Initial Algorithm developed from the test set of the Development Cohort was deployed against the remaining 7020 charts flagged in our screening tool to create the Refinement Cohort which contained 741 additional predicted PAH cases. Among the 741 predicted cases, we identified an additional 468 manually confirmed cases of PAH. Identification of these additional 468 cases allowed more robust training and testing of the algorithm in the next step.

### Final development of algorithm

The Development and Refinement Cohorts were combined to become the Final Cohort, which included 653 PAH cases and 1041 confirmed non-cases. This cohort was divided into two groups: a training set, which included 80% of the patients (n = 1356) and a test set, which included the other 20% of the patients (n = 338). The RF algorithm performed slightly better than the other methods in the training and test sets. The RF algorithm yielded an area under the curve of 0.94 (95% [CI] 0.94–0.95, sensitivity 0.85 (95% [CI] 0.83–0.87), specificity 0.92 (95% [CI] 0.91–0.92), PPV of 0.87 (95% [CI] 0.85–0.88), and NPV 0.91 (95% [CI] 0.91–0.92) in the training set (Table 1). The algorithm derived from the training set of Cohort C was considered our Final Algorithm. In our last step, the performance of the final algorithm was examined against the test set of the Final Cohort. The final RF algorithm yielded an area under the curve (AUC) of 0.96, sensitivity of 0.88, specificity of 0.93, positive predictive value (PPV) of 0.89, and negative predictive value (NPV) of 0.92 in the test set (Table 1). Final results for the additional machine learning techniques are reported in Additional file 1: Table S3.

**Table 1:** Test characteristics of the RF algorithm for the Testing Algorithm in the Final Cohort

Dataset	AUC	Sensitivity	Specificity	PPV	NPV
Test (n = 338)	0.96	0.88	0.93	0.89	0.92
Training (n = 1356)	0.94 (0.94–0.95)	0.85 (0.83–0.87)	0.92 (0.91–0.92)	0.87 (0.85–0.88)	0.91 (0.91–0.92)

For the cohort that was split into training and test sets (labeled “Final Test Set” and “Final Training Set” in Fig. 1). Values for training represent means and 95% confidence intervals based on 30 samples from tenfold cross validation repeated 3 times

### Features of machine learning algorithm

**Fig. 3:**
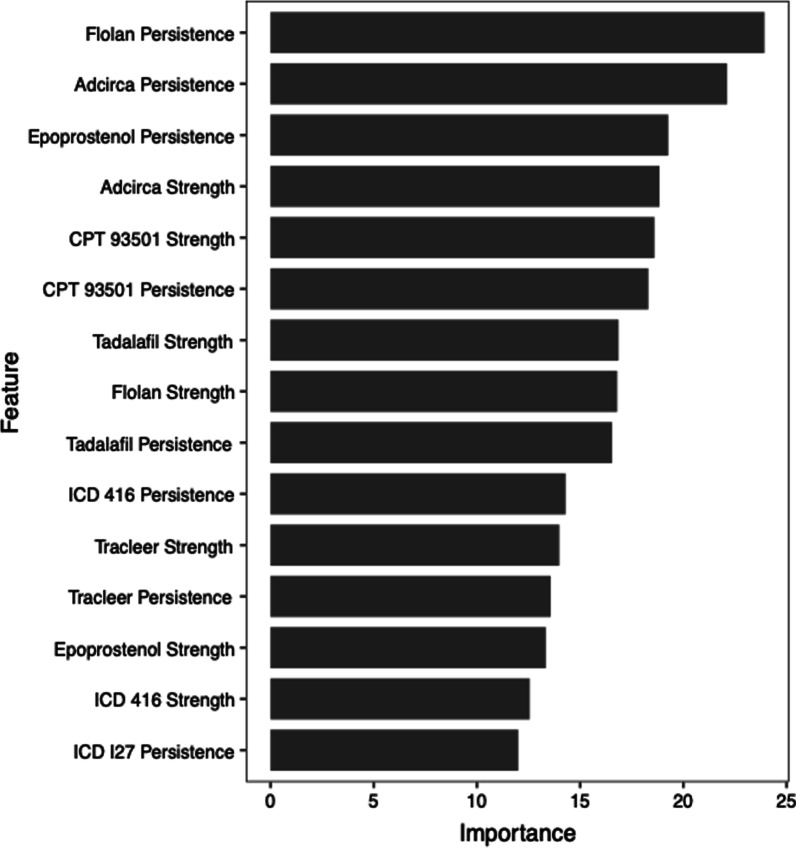
Ranked features of final random forests algorithm. Importance of features of the algorithm are depicted. Strength was defined as the number of mentions throughout the medical record. Persistence was defined as the number of days that code or term stayed on the record. Durability was defined as the persistence divided by the length of the record after the first appearance on the record

The features that contributed most to the performance of the final random forest algorithm are shown in Fig. 3. The distribution of strength, persistence, and durability for each of the final model variables is shown in Additional file 1: Fig. S2) The top features include persistence of the medication Flolan, the persistence of the medication Adcirca, the persistence of the medication Epoprostenol the strength of the medication Adcirca, and the strength of the CPT code 93501 for right heart catheterization.

### Internal validation of random forest algorithm in the synthetic derivative

We next tested the hypothesis that our Final Algorithm would identify a patient population with demographic and clinical characteristics similar to published PAH registries. This experiment simulates real world application of the Final Algorithm in a claims-based database in that it only uses administrative data and we did not manually confirm PAH cases. We deployed the final RF algorithm across Vanderbilt’s entire medical system using the SD (approximately 2.5 million unique records), excluding PAH cases and non-cases used to develop the algorithm. The algorithm identified an additional 137 cases of suspected PAH and these were added to 128 cases from the test set of the Final Cohort which were not used to train the algorithm. These cases were not manually reviewed (as would be the case in an administrative database) but instead aggregated to compare characteristics of these patients to non-cases in the SD and to PAH patients in prior registries (Table 2). The group of patients identified as PAH by the algorithm fit traditional demographics of PAH with a mean age of 52 ± 14 years, a predominance of females (72%), and high prevalence of connective tissue disease (33%) and congenital heart disease (19%) in identified patients. Further, in those patients with hemodynamic data (189/265), we found evidence of predominantly pre-capillary pulmonary hypertension with a mean pulmonary artery pressure of 49.8 ± 13.6 mmHg, pulmonary capillary wedge pressure of 11.3 ± 6.1 mmHg, and pulmonary vascular resistance of 9.9 ± 5.9 Wood Units.

**Table 2:** Test characteristics of cases identified by the random forest algorithm when deployed on the Synthetic Derivative (including cases from the test set)

Test Characteristics	RF Algorithm in SD	RF Algorithm SD Non-Cases	French Registry	REVEAL	UK-Ireland Registry
n	265	2,270,971	674	2525	482
Age (years)	52.0 (13.7)	52.0 (21.4)	50.0 (15.0)	50.1 (14.4)	50.1 (17.1)
Sex, % female (n)	72.1% (191)	54.2% (1,253,660)	65.3%	79.5%	69.9%
*Comorbidities*					
CTD (n)	33.2% (88)	2.0% (465)	15.3%	25%	–
CHD (n)	19.2% (51)	0.9% (19,950)	11.3%	10%	–
*Medications*					
ERA (n)	70.6% (187)	< 0.1% (26)	–	47%	44.2%
GS stimulators (n)	1.5% (4)	< 0.1% (9)	–	–	–
PDE5 inhibitors (n)	84.2% (223)	0.4% (9,224)	–	49%	29.2%
Prostanoids (n)	63.0% (167)	< 0.1% (2)	–	–	18.8%
*Hemodynamics *(n = 189)		(n = 8806)			
RA pressure (mmHg)	10.1 (6.0)	8.8 (8.3)	8 (5.)	9.3 (5.6)	10.1 (6.0)
Mean PA (mmHg)	49.8 (13.6)	27.7 (11.7)	55 (15)	50.7 (13.6)	54.1 (13.9)
PWP (mmHg)	11.3 (6.1)	15.5 (8.3)	8.0 (3)	9.1 (3.5)	9.2 (3.5)
CO, Fick (L/min)	4.8 (2)	5.6 (3.7)	–	–	4.0 (1.5)**
CO, TD (L/min)	4.8 (1.7)	5.1 (2.4)	–	–	4.0 (1.5)**
CI, Fick (L/min/M^2^)	2.5 (1.0)	2.9 (2.7)	2.9 (0.9)*	2.4 (0.8)*	2.1 (6.3)
CI, TD (L/min/M^2^)	2.5 (0.8)	2.6 (2.2)	2.9 (0.9)*	2.4 (0.8)*	2.1 (6.3)
PVR (Wood units)	9.9 (5.9)	2.6 (2.5)	***	***	12.8 (6.3)

*SD*  synthetic derivative, *CTD*  connective tissue disease, *CHD*  congenital heart disease, *ERA*  endothelin receptor antagonists, *GC*  guanylate cyclase, *PDE5*  phosphodiesterase type 5, *RA*  right atrial, *PA*  pulmonary arterial, *PWP*  pulmonary wedge pressure, *CO*  cardiac output, *TD*  thermodilution, *CI*  cardiac index, *PVR*  pulmonary vascular resistance. Data expressed as % (n) or mean (SD) unless otherwise noted. For French Registry [32], all values taken from Table 1. For REVEAL [34], demographics and hemodynamics are taken from Table 1, and medications are taken from Table 2. For UK-Ireland Registry [33], demographics and hemodynamics are taken from Table 1, and medications are taken from Table 2 (values added across all years, n = 479)

^*^For REVEAL, Fick CI was used unless it was missing, in which case thermodilution CI was used. For French Registry, CI method was not indicated

^**^Method not indicated

^***^PVRI was reported for French Registry (*M* = 20.5, *SD* = 10.2) and REVEAL (*M* = 21.1, *SD* = 12.5)

## Discussion

Recognizing an unmet need to confidently identify PAH in health care systems across the country and claims databases, we used machine learning tools to develop and validate an algorithm that identifies patients with PAH with strong testing characteristics. We further tested the algorithm in a second cohort within the SD and showed that cases it identifies share demographic and hemodynamic similarities to published PAH registries [21, 22, 32]. Importantly, our algorithm only required administrative data (CPT or ICD 9/10 codes and medications used for PAH), yet improved on previously published CB-algorithms by incorporating strength and persistence of these codes, and thus this algorithm could be useful in claims-based databases. We employed a modified active learning process to develop this algorithm that identified subjects with true disease in an initial screen which was used to develop a model that was then refined and tested on larger cohorts. The final model was deployed on the entire EMR (excluding patients used to develop the algorithm), in a form of split cohort in silico validation. Overall this approach demonstrated the ability of this algorithm to detect individuals in the EMR with disease characteristics similar to PAH.

There are many challenges in the care and diagnosis of PAH that would benefit from algorithms to detect this disease in claims-based algorithms. First, risk factors for idiopathic PAH other than use of anorexigens [33], have been difficult to detect and subject to recall bias. Using a claims-based dataset, preceding exposures and patient characteristics could be explored. Real-world efficacy of many PAH therapies and combination therapies could be tested and effects of non-PAH therapeutics and demographic features on PAH outcomes could be assessed, as they have been in undifferentiated populations with pulmonary hypertension [34, 35]. Thus far other algorithms have not achieved this level of confidence in PAH cases and thus the impact of their findings regarding this specific diagnosis are limited [4, 7, 12, 15, 36].

PAH is a diagnosis of exclusion, requiring complex cardiopulmonary assessment including right heart catheterization and treated with relatively few medications. A major challenge to identification of cases in EMRs is lack of precision in ICD9/10 codes as they are generally not reflective of current classification of pulmonary hypertension [5, 7]. Prior attempts at development of algorithms have had challenges including lack of access to primary, little or no patient-level data for manual chart review or missing validation in a second cohort [4, 14, 15, 36]. We were able to overcome these limitations using the SD which can be searched both by simple ICD9/10 and CPT codes and medication and also contains granular diagnostic data to positively identify PAH cases who are referred to our high-volume center for pulmonary hypertension assessment. After screening the entire SD by these minimal features, we manually reviewed 1694 charts and assigned patients to “PAH” or “Not PAH” status, which trained the machine learning algorithm. The process of using known cases to train an algorithm is a departure from prior studies in this area which have created algorithms a priori (e.g. two codes plus one medication) and then reviewed charts to determine their performance. By “feeding” the algorithm known cases upfront and using a multi-step training process, we were able to develop an algorithm that outperforms prior efforts. As previously hypothesized, the use of machine learning made separates our algorithm from those before it. For comparison, the best PPV from the Papani et al. algorithms was 69.4% [12] and from the Gillmeyer et al. algorithms was 86% [14]. Our AUC of 0.96 compares favorably to the c-statistic of 0.87 cited by Papani et al. [12].

A strength of our study is performing a functional “test” of the algorithm in a different, unstudied portion of the SD. Our final experiment simulated real world application of the algorithm to an external dataset or EMR. When we applied the algorithm to our entire EMR, excluding all patients used in the training stages, we identified 265 patients with characteristics similar to published PAH registries [21, 22, 32] including high prevalence of congenital heart disease and connective tissue disease, female predominance and, in those with right heart catheterization data, hemodynamics consistent with precapillary PH [5]. It is important to note that these 265 cases identified by the machine learning algorithm were not included in the derivation and validation steps. In other words, the algorithm never saw these cases while it was being trained. Specifically, the final test set from Cohort C contained 338 subjects total. Of these 338, there were 137 predicted cases by the RF algorithm. The RF was deployed on the entire SD and predicted 128 remaining cases that were not in the Final Cohort. Thus, the RF algorithm was not trained on the 265 cases identified in the EHR. While the prevalence of 265/2,500,000 may be somewhat higher than the estimated prevalence of PAH in the US population [32], our center is a referral center for PAH, cardiovascular and rheumatologic disease that may be driving this finding.

Our study used machine learning algorithms to improve on simple binary use of code or medication. We explored three different machine learning algorithms: Elastic net [24], RF [26], extreme gradient boosting (XGBoost) [26]. These applications have been applied to predict urinary tract infections [37], chronic kidney disease [38], survival in systolic heart failure [39], and mortality in postmenopausal women [40]. All of the machine learning algorithms used here have hyperparameters that must be set prior to training. The performance of the algorithm is often highly dependent on the choice of the hyperparameters. Therefore, hyperparameters must be tuned in order to gain the best performance possible from the model. Here, we utilized Bayesian optimization [27], a type of black-box optimization technique aimed at globally optimizing over the parameter space of costly functions with greater efficiency than other methods such as grid search. In our study, optimization was carried out over the training set by maximizing the average area under the curve resulting from stratified tenfold (3-times-repeated) cross validation. After finding the optimal hyperparameters, the final algorithm (used for testing) was trained on the entire training set. Although all three machine learning algorithms performed well, we ultimately tested the RF algorithm on the entire remaining SD because of its slightly superior testing characteristics. RF utilizes an ensemble learning method known as bagging in which classifiers are trained using bootstrapped samples of the data and a majority vote is taken to produce the final result.

Our work is not without limitations. One potential source of error for the algorithm is inappropriate use of PAH-specific medications. We attempted to address this through extensive manual chart review of large numbers of suspected PAH. While cases of mixed etiology PH were possible, if the reviewing expert and treating clinician determined that the patient had enough features of PAH to treat the patient as such, they were included in the true cases. No algorithm can account for the current limitations of classification of pulmonary hypertension and algorithms such as this for rare diseases are at risk of class imbalance. As previously suggested, alignment of ICD codes with the WSPH definitions of pulmonary hypertension would like improve coding and performance of this algorithm alike [4]. While we performed an internal validation of the algorithm in a new, unstudied portion of the SD, this algorithm will be strengthened through validation in non-referral centers with a lower prevalence of the disease. Our CB approach is only useful in countries that use similar data. And lastly, the elevated prevalence of PAH at our center compared to the general population may reduce the generalizability and external validity of our algorithms.

## Conclusions

In conclusion, we created and validated a machine learning algorithm that identified specifically PAH patients from the EMR at a tertiary referral center. This algorithm performed with favorable testing characteristics. When deployed across an entire medical system, identified case demographic and clinical features were similar to known PAH patients from previously studied registries.

## Supplementary Information

**Additional file 1: Figure S1.** Feature importance for XGBoost and Elastic Net. **Figure S2. **Random forest importance values for each variable and associated variation (strength, durability, and persistence). **Table S1.** Number of subjects with each variable and percentage of Synthetic Derivative (N = 2,278,297). **Table S2.** Test characteristics of all three algorithms for the Development Cohort. Training results show mean AUC and 95% confidence intervals for each model based on 3 times repeated 10-fold cross validation (PPV and NPV were not computed during training). **Table S3.** Test characteristics of all non-RF algorithms for the Final Cohort. Training results show mean AUC and 95% confidence intervals for each model based on 3 times repeated 10-fold cross validation.

## Data Availability

The datasets generated during this study are available from the corresponding author on reasonable request.

## Abbreviations

AUC: Area under the curve
EMR: Electronic medical record
NPV: Negative predictive value
PAH: Pulmonary arterial hypertension
PH: Pulmonary hypertension
PPV: Positive predictive value
RF: Random forest
SD: Synthetic derivative
WSPH: World Symposium on Pulmonary Hypertension
