# Supplementary material for: An algorithm to identify cases of pulmonary arterial hypertension from the electronic medical record
Source: Respir Res. 2022 May 28;23:138. doi: 10.1186/s12931-022-02055-0 (PMC9145474; doi:10.1186/s12931-022-02055-0)
Supplement: Supplementary file 1 — Additional file 1: Figure S1. Feature importance for XGBoost and Elastic Net. Figure S2. Random forest importance values for each variable and associated variation (strength, durability, and persistence). Table S1. Number of subjects with each variable and percentage of Synthetic Derivative (N = 2,278,297). Table S2. Test characteristics of all three algorithms for the Development Cohort. Training results show mean AUC and 95% confidence intervals for each model based on 3 times repeated 10-fold cross validation (PPV and NPV were not computed during training). Table S3. Test characteristics of all non-RF algorithms for the Final Cohort. Training results show mean AUC and 95% confidence intervals for each model based on 3 times repeated 10-fold cross validation. [file 12931_2022_2055_MOESM1_ESM.docx]

**ADDITIONAL MATERIAL**

**Methods:**

*Elastic Net and XGBoost*

We selected two other popular machine learning algorithms in order to compare to the RF algorithm: Elastic net (Zou & Hastie, 2005) and Extreme gradient boosting (XGBoost). Elastic net is a penalized regression method that can be viewed as a combination of lasso regression (Tibshirani, 1996), which penalizes according to the L_1_-norm of the coefficients, and ridge regression (Hoerl & Kennard, 1988), which penalizes according to the L_2_-norm. XGBoost is an ensemble learning methods in which many classifiers (in this case, decision trees) are trained and their results aggregated. XGBoost uses an ensemble learning technique known as gradient boosting in which classifiers are trained in succession with each new classifier trained on the residuals of the prior classifier. Model training and testing was identical to that used for RF. Results for training and test are shown in Table A1 and A2, respectively. Feature importance is shown in Figure A1.

*Training details for all models:*

Models were tuned on the training sets with cross validation (10-fold 3-times-repeated).

*Optimization:*

Optimization was carried out over the training set by maximizing the average area under the curve resulting from stratified 10-fold (3-times-repeated) cross validation. After finding the optimal hyperparameters, the final algorithm (used for testing) was trained on the entire training set. All algorithms were trained and tested in the R programming language.

**Figure S1.** Feature importance for XGBoost and Elastic Net.

**Figure S2.** Random forest importance values for each variable and associated variation (strength, durability, and persistence).


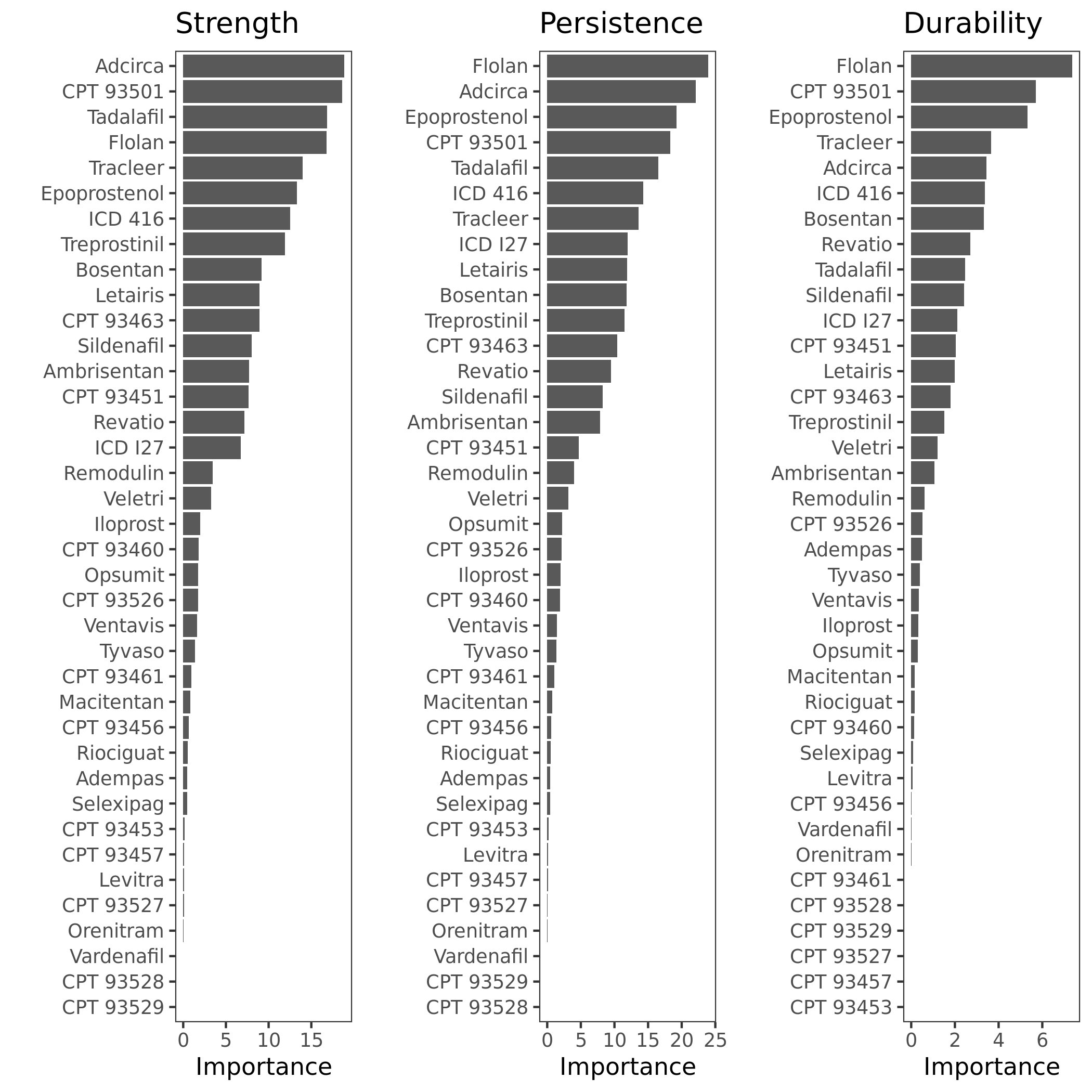


**Table S1**. Number of subjects with each variable and percentage of Synthetic Derivative (N = 2,278,297).

| **Variable** | **N (%)** |
| --- | --- |
| ICD 416 (Primary pulmonary hypertension) | 17478 (0.77) |
| Sildenafil | 17056 (0.75) |
| Levitra | 6462 (0.28) |
| ICD I27 | 5850 (0.26) |
| Tadalafil | 5742 (0.25) |
| CPT 93460 (coronary angiography with right and heart catheterization) | 5262 (0.23) |
| CPT 93526 (right and left heart catherization) | 4607 (0.2) |
| Epoprostenol | 4274 (0.19) |
| CPT 93451 (right heart catheterization) | 4260 (0.19) |
| Revatio | 4008 (0.18) |
| CPT 93501 (right heart catheterization) | 3098 (0.14) |
| Flolan | 2318 (0.1) |
| CPT 93463 (right heart catheterization with pharmacologic administration) | 1760 (0.08) |
| Vardenafil | 1733 (0.08) |
| CPT 93456 (coronary angiography with right heart catheterization) | 1036 (0.05) |
| CPT 93461 (right and left heart catheterization with coronary and bypass graft angiography) | 909 (0.04) |
| Adcirca | 653 (0.03) |
| Tracleer | 440 (0.02) |
| Bosentan | 396 (0.02) |
| Letairis | 349 (0.02) |
| Veletri | 293 (0.01) |
| Ambrisentan | 277 (0.01) |
| Treprostinil | 271 (0.01) |
| CPT 93457 (right heart catheterization with coronary and bypass graft angiography) | 232 (0.01) |
| CPT 93453 (right and left heart catheterization with left ventriculography) | 227 (<.01) |
| Iloprost | 181 (<.01) |
| Opsumit | 163 (<.01) |
| Remodulin | 138 (<.01) |
| Tyvaso | 128 (<.01) |
| CPT 93527 (Combined right heart catheterization and transseptal left heart catheterization through intact septum) | 124 (<.01) |
| Macitentan | 108 (<.01) |
| Ventavis | 100 (<.01) |
| Adempas | 59 (<.01) |
| Selexipag | 59 (<.01) |
| Riociguat | 56 (<.01) |
| CPT 93529 (Combined right heart catheterization and left heart catheterization through existing septal opening) | 16 (<.01) |
| Orenitram | 10 (<.01) |
| CPT 93528 (Combined right heart catheterization with left ventricular puncture) | 2 (<.01) |

**Table S2**. Test characteristics of all three algorithms for the Development Cohort. Training results show mean AUC and 95% confidence intervals for each model based on 3 times repeated 10-fold cross validation (PPV and NPV were not computed during training).

| Algorithm | AUC | Sensitivity | Specificity | PPV | NPV |
| --- | --- | --- | --- | --- | --- |
| Test |  |  |  |  |  |
| RF | 0.91 | 0.66 | 0.98 | 0.88 | 0.92 |
| Elastic Net | 0.87 | 0.45 | 0.98 | 0.84 | 0.88 |
| XGBoost | 0.94 | 0.78 | 0.94 | 0.76 | 0.94 |
| Training |  |  |  |  |  |
| RF | 0.91  (0.89-0.93) | 0.63  (0.59-0.68) | 0.97  (0.95-0.98) |  |  |
| Elastic Net | 0.873  (0.85-0.89) | 0.50  (0.45-0.55) | 0.91  (0.90-0.93) |  |  |
| XGBoost | 0.92  (0.91-0.94) | 0.76  (0.72-0.81) | 0.98  (0.97-0.98) |  |  |

**Table S3**. Test characteristics of all non-RF algorithms for the Final Cohort. Training results show mean AUC and 95% confidence intervals for each model based on 3 times repeated 10-fold cross validation.

| **Algorithm** | **AUC** | **Sensitivity** | **Specificity** | **PPV** | **NPV** |
| --- | --- | --- | --- | --- | --- |
| **Test** |  |  |  |  |  |
| XGBoost | 0.96 | 0.88 | 0.93 | 0.88 | 0.92 |
| Elastic Net | 0.93 | 0.65 | 0.94 | 0.88 | 0.81 |
| **Training** |  |  |  |  |  |
| XGBoost | 0.95  (0.94-0.95) | 0.86  (0.85-0.88) | 0.93  (0.92-0.94) | 0.87  (0.86-0.89) | 0.91  (0.90-0.92) |
| Elastic Net | 0.92  (0.91-0.93) | 0.72  (0.70-0.74) | 0.91  (0.90-0.93) | 0.86  (0.85-0.88) | 0.84  (0.83-0.85) |

**References**

Chen, T., & Guestrin, C. (2016). XGBoost: A scalable tree boosting system. *Proceedings of the ACM SIGKDD International Conference on Knowledge Discovery and Data Mining*, *13*-*17*-*Augu*, 785–794. https://doi.org/10.1145/2939672.2939785

Chen, T., He, T., Benesty, M., Khotilovich, V., Tang, Y., Cho, H., … Li, Y. (2019). *xgboost: Extreme Gradient Boosting*. Retrieved from https://cran.r-project.org/package=xgboost

Friedman, J., Hastie, T., & Tibshirani, R. (2010). Regularization paths for generalized linear models via coordinate descent. *Journal of Statistical Software*, *33*(1), 1–22. Retrieved from http://www.jstatsoft.org/v33/i01/

Hoerl, A., & Kennard, R. (1988). Ridge regression. In *Encyclopedia of Statistical Sciences* (pp. 129–136). New York: Wiley.

Kuhn, M. (2020). *caret: Classification and regression training*. Retrieved from https://cran.r-project.org/package=caret

Tibshirani, R. (1996). Regression shrinkage and selection via the lasso. *Journal of the Royal Statistical Society: Series B (Methodological)*, *58*(1), 267–288.

Wright, M. N., & Ziegler, A. (2017). ranger: A Fast Implementation of Random Forests for High Dimensional Data in C++ and R. *Journal of Statistical Software*, *77*(1), 1–17. https://doi.org/10.18637/jss.v077.i01

Zou, H., & Hastie, T. (2005). Regularization and variable selection via the elastic net. *Journal of the Royal Statistical Society: Series B*, *67*(2), 301–320.
